# Elmira Academy of Medicine

**Published:** 1912-04

**Authors:** 


### SOCIETY PROCEEDINGS

---

### Elmira Academy of Medicine

At the regular meeting of the Elmira Academy of Medicine, hold on March 6, 1912, the following amendment to the fee bill was unanimously adopted.

"We recommend the adoption of an amendment to the fee bill making the minimum charge for an examination in lunacy $15.00 and the minimum charge for an autopsy $20.00, for each physician.

We also recommend that the Board of Supervisors be notified of this action.

---

The Elmira Academy of Medicine will hold its regular meeting on April 3, 1912, at 8.15 P. M., in the Society rooms, at the Federation Building.

Program—"The Relation of Immunity to Serum Therapy," LaRue Colgrove, M. D.; "My Experience with Salvarsan," N. H. Soble, M. D.

Herbert W. Fudge, M. D., President.

Charles Haase, M. D., Secretary.

---

Dr. A. L. Benedict and Dr. R. Montfort Schley entertained the Buffalo Association, Sons of the Revolution, at the University Club, on Thursday evening, March 28th, 1912.

Mr. Robert M. Codd, Jr., B S—LL.B., spoke on "The King's Loyal Americans."

---

The Buffalo Association of the University of Rochester Alumni numbers amongst its members the following physicians residing in Buffalo and vicinity: Dr. George R. Stearns, Class of 1875, Dr. William H. Thornton Class of 1879, Dr. J. W. LeSeur of Batavia, Class of 1882, Dr. A. L. Benedict, Class of 1887 (but graduated at Michigan), Dr. Herman K. DeGroat, Class of 1892; Dr. L. Kauffman, Class of 1896. Dr. Benedict was elected second vice-president and Dr. Kauffman secretary for the ensuing year.

